# Advanced Electrospun Chitosan-(Polylactic Acid)-(Silver Nanoparticle)-Based Scaffolds for Facilitated Healing of Purulent Wounds: A Preclinical Investigation

**DOI:** 10.3390/polym17162225

**Published:** 2025-08-15

**Authors:** Yevhen Samokhin, Yuliia Varava, Anna Butsyk, Roman Moskalenko, Yevheniia Husak, Bohdan Dryhval, Valeriia Korniienko, Ihor Zhyvotovskyi, Vyacheslav Kukurika, Artem Shmatkov, Agne Ramanaviciute, Rafal Banasiuk, Maksym Pogorielov, Arunas Ramanavicius, Viktoriia Korniienko

**Affiliations:** 1 Biomedical Research Centre, Medical Institute, Sumy State University, 116, Kharkivska St., 40007 Sumy, Ukraine; justinsamokhin@gmail.com (Y.S.); yuliia.varava@gmail.com (Y.V.); drigval007@gmail.com (B.D.); kornienvaleria18@gmail.com (V.K.); i.zhivotovsky@med.sumdu.edu.ua (I.Z.); kukurikav25@gmail.com (V.K.); artemshmatkoff@gmail.com (A.S.); maksym.pogorielov@lu.lv (M.P.); 2 Ukrainian-Swedish Research Center SUMEYA, Medical Institute, Sumy State University, 116, Kharkivska St., 40007 Sumy, Ukraine; a.butsyk@med.sumdu.edu.ua; 3 Department of Pathology, Medical Institute, Sumy State University, 116, Kharkivska St., 40007 Sumy, Ukraine; r.moskalenko@med.sumdu.edu.ua; 4 Faculty of Chemistry, Silesian University of Technology, 44-100 Gliwice, Poland; yevheniia.husak@polsl.pl; 5 Department of Nanotechnology, Centre for Physical Sciences and Technology, Sauletekio Av. 3, LT-10257 Vilnius, Lithuania; agne.ramanaviciute@gmail.com; 6 Department of Physical Chemistry, Institute of Chemistry, Faculty of Chemistry and Geosciences, Vilnius University, Naugarduko Str. 24, LT-03225 Vilnius, Lithuania; 7 NanoWave Ltd., 02-676 Warsaw, Poland; banasiuk@gmail.com; 8 Institute of Atomic Physics and Spectroscopy, University of Latvia, 3 Jelgavas St., LV-1004 Riga, Latvia

**Keywords:** electrospinning, chitosan, polylactic acid, silver nanoparticles, purulent wounds, wound healing, antimicrobial biomaterials

## Abstract

Biomaterials modified by antibacterial substances, including nanoparticles, open new opportunities for the effective treatment of infected wounds. Unfortunately, most publications focused only on experiments in vitro, with limited understanding of their potential for the clinic. This study evaluates the effectiveness in vivo of electrospun chitosan/polylactic acid (Ch/PLA) membranes enriched with silver nanoparticles (AgNPs) for purulent wound treatment. The composite biomaterial integrates chitosan’s biocompatibility and antimicrobial activity with PLA’s structural integrity, while AgNPs enhance antibacterial efficacy against major wound pathogens, including *Staphylococcus aureus*, *Pseudomonas aeruginosa*, and *Escherichia aureus*. A full-thickness purulent wound model was established in a rat model, and the animals were divided into three treatment groups: (i) Ch/PLA, (ii) Ch/PLA-AgNPs, and (iii) PLA-chlorhexidine (control). Wound healing was monitored over 21 days through macroscopic evaluation, histology, immunohistochemistry, and microbiological analysis. The Ch/PLA-AgNPs membranes significantly reduced bacterial colonization within 4–6 days, promoted granulation tissue formation, and accelerated epithelialization compared to the non-modified Ch/PLA scaffold. By day 15, complete wound closure was observed in the Ch/PLA-AgNPs group, comparable to PLA-chlorhexidine-treated wounds. Immunohistochemical analysis revealed a controlled inflammatory response with a balanced macrophage M1/M2 transition, supporting efficient tissue regeneration. Furthermore, systemic toxicity assessments indicated no significant adverse effects on internal organs. These findings demonstrate that electrospun Ch/PLA-AgNPs membranes effectively accelerate purulent wound healing by combining antimicrobial protection with biocompatible tissue support. This innovative approach presents a promising alternative to conventional wound dressings and paves the way for clinical applications in managing infected wounds.

## 1. Introduction

The development of effective biomaterials for wound healing presents significant challenges during their transition from in vitro investigations to applications in vivo [1,2]. This issue becomes particularly pronounced in the case of materials designed for infected wounds, where the complex interplay of microbial contamination, immune response, and tissue regeneration demands a high level of functional specificity. In vitro studies often fail to fully replicate the intricate biological environment of infected wounds, leading to gaps in understanding how these materials will perform in clinical or preclinical settings [3,4]. For instance, purulent wounds are characterized by heightened bacterial load and inflammatory responses, which require biomaterials to possess not only biocompatibility and mechanical stability but also potent antimicrobial properties to facilitate effective healing [5,6]. Addressing these challenges necessitates innovative approaches, such as the integration of antimicrobial agents like silver nanoparticles within biocompatible polymer matrices [7,8], to bridge the gap between laboratory findings and clinical efficacy.

Purulent wounds represent a significant challenge in healthcare, posing serious risks to patient outcomes and imposing a considerable financial burden on healthcare systems worldwide [9]. These wounds are characterized by the presence of infection, inflammation, and necrotic tissue, which hinder the natural healing process and often lead to prolonged hospital stays, repeated interventions, and an increased likelihood of complications, including systemic infections like sepsis [10,11,12]. Infections associated with purulent wounds are particularly concerning in an era of rising antimicrobial resistance, further complicating treatment and recovery [13]. From a societal perspective, chronic and infected wounds disproportionately affect vulnerable populations, including the elderly, individuals with diabetes, and those with compromised immune systems [14,15,16]. Economically, the costs associated with the management of these wounds are staggering, including expenses for advanced wound dressings, antibiotics, surgical interventions, and extended care. In the United States and Europe alone, the annual expenditure on wound care exceeds tens of billions of dollars, highlighting the urgent need for innovative, cost-effective solutions to address this pervasive healthcare issue [17].

Microbial colonization plays a central role in the pathophysiology of purulent wounds, with bacterial biofilms representing a critical barrier to effective treatment [18]. These biofilms, which consist of microbial cells embedded in a self-produced extracellular matrix, adhere to wound surfaces and create a protective environment that enhances bacterial survival and resistance to both the host immune response and conventional antibiotics [19]. Common pathogens, including *Staphylococcus aureus*, *Pseudomonas aeruginosa*, and *Escherichia coli*, are frequently implicated in these infections and exhibit high levels of antibiotic resistance when embedded in biofilms [20]. This resistance not only limits the efficacy of standard antimicrobial therapies but also contributes to the global health crisis of rising antibiotic resistance, which the World Health Organization has identified as one of the most pressing threats to human health [21]. As biofilms prolong inflammation and hinder tissue regeneration, they aggravate wound chronicity and increase the risk of systemic infections. These challenges underscore the urgent need for alternative treatments that target biofilm-associated infections. Innovative approaches, such as the use of biomaterials integrated with antimicrobial nanoparticles [22], provide a promising solution by combining physical support for wound healing with enhanced antimicrobial efficacy, thereby addressing the limitations of traditional therapies [19,23].

Biomaterial production integrates principles of polymer science, nanotechnology, and bioengineering to fabricate structures that mimic the extracellular matrix and provide an optimal environment for cell proliferation and tissue regeneration [24]. Traditional methods, such as solvent casting [25], freeze-drying [26,27], and 3D printing [28,29], have been widely used to produce biomaterials with defined architectures and compositions. However, these techniques often lack the ability to produce nanostructures that replicate the intricate features of the native ECM, a limitation that hinders their full potential in regenerative applications. Electrospinning has emerged as a transformative technology in biomaterial production, offering unparalleled control over fiber morphology, composition, and functionalization [30]. Electrospun scaffolds can be fabricated from a wide range of materials, including natural polymers (e.g., chitosan, collagen, and hyaluronic acid) [31,32] and synthetic polymers (e.g., polylactic acid [PLA], polycaprolactone [PCL]) [33,34,35], and polyethylene glycol [PEG]). Additionally, the process allows for the incorporation of bioactive molecules, such as growth factors, antibiotics, or nanoparticles, to enhance specific functionalities like antimicrobial activity or cell signaling [36,37].

Polymer blends or multilayered electrospun scaffolds have demonstrated superior results in wound healing, as each polymer can play a unique role in the regeneration process [38,39,40]. In our research, we selected polylactic acid (PLA) as a biocompatible supportive material due to its ability to maintain membrane integrity, facilitate exudate sorption, and provide adequate oxygen access to the wound bed [41,42]. For the second layer of our bilayered scaffold, we chose chitosan, a natural polymer known for its antimicrobial properties and ability to promote tissue regeneration [43]. This combination of PLA and chitosan offers a synergistic approach, with PLA providing structural support and chitosan actively contributing to infection control and cellular activities essential for wound closure. The bilayered architecture of the scaffold ensures both functional and mechanical balance, optimizing the wound-healing environment. Despite numerous reports highlighting the strong antibacterial effects of chitosan [44,45], it remains insufficiently effective against complex microbial associations typically found in infected wounds [46]. To enhance the antibacterial potential of Ch/PLA electrospun membranes, we incorporated silver nanoparticles (AgNPs) into the material. In our previous research, this composite demonstrated remarkable effectiveness against major wound pathogens, including *Staphylococcus aureus*, *Pseudomonas aeruginosa*, and *Escherichia coli*, while maintaining high biocompatibility [47,48].

In this study, we aimed to evaluate the effectiveness in vivo of the novel Ch/PLA-AgNPs electrospun membranes. This included detailed monitoring of bacterial elimination, the regenerative process in infected wounds, and the systemic toxicity of the biomaterial. Histological assessment of the wound repair process was performed by evaluation of inflammation and healing markers MPO, CD68, and CD163. The comprehensive in vivo assessment presented in this research not only underscores this innovative technology’s antimicrobial efficacy and regenerative potential but also addresses critical safety concerns, thereby bringing this advanced biomaterial closer to clinical application and the medical market.

## 2. Materials and Methods

### 2.1. Materials

Ch/PLA and Ch/PLA-AgNPs electrospun membranes were prepared as described in our previous research [48]. Chitosan (low molecular weight powder, 890,000 Da) was purchased from GlenthamLife Sciences in Corsham, UK (CAS 9012-76-4). The electrospun Ch/PLA membranes were functionalized with silver nanoparticles (AgNPs) at a concentration of 100 µg/mL supplied by Nano Pure Co., Wrocław, Poland. All media (Muller–Hinton agar (#70191), Muller–Hinton broth (#70192), MacConkey agar #70143, mannitol-salt agar #63567, and cetrimide agar #1.05284 Merck Millipore (Burlington, MA, USA)) were purchased from Sigma-Aldrich (Darmstadt, Germany) and used without further modification. Chlorhexidine solution 0.05% was obtained from “Fitofarm”, Ukraine. Bacteriological experiments were conducted on *S. aureus* (*S. aureus*, B 918) and *Escherichia coli* (*E. coli*, B 926), and *Pseudomonas aeruginosa* (*P. aeruginosa*, ATCC 27853) obtained from the Ukrainian Collection of Microorganisms (UCM) of D.K. Zabolotny Institute of Microbiology and Virology of the National Academy of Sciences of Ukraine (IMV NASU).

### 2.2. Electrospun Patches Structural Assessment

Membrane samples, each 0.5 cm^2^, were observed using scanning electron microscopy (SEM) (Phenom ProX, Phenom-World BV, Eindhoven, The Netherlands), which was equipped with an energy-dispersive X-ray spectrometer (EDX) to prove the morphological and chemical features. The average fiber diameter and porous area fraction were evaluated with the Fiji software (ImageJ version 1.51f; Java version 1.8.0_102) [48].

### 2.3. Experimental Animals

Fifty-four male Wistar rats, aged 12 weeks and weighing between 200 and 220 g, were obtained from the Vivarium of Sumy State University (Ukraine) for use in this experiment. The animals were individually housed in standard cages under controlled environmental conditions, maintained at an ambient temperature of 25 °C with a 12 h light/dark cycle. All rats had unrestricted access to food and water but were fasted for 12 h prior to the experimental procedures. The study protocols were reviewed and approved by the Commission on Bioethics Compliance in Experimental and Clinical Research at Sumy State University. All procedures adhered strictly to Directive 2010/63/EU of the European Parliament and of the Council on the Protection of Animals Used for Scientific Purposes, ensuring ethical treatment and care (Commission on Bioethics Compliance in Experimental and Clinical Research of Sumy State University, Protocol #2/04 dated 9 April 2024).

### 2.4. Rat Skin Defect Model and Treatment Protocol

**Figure 1:**
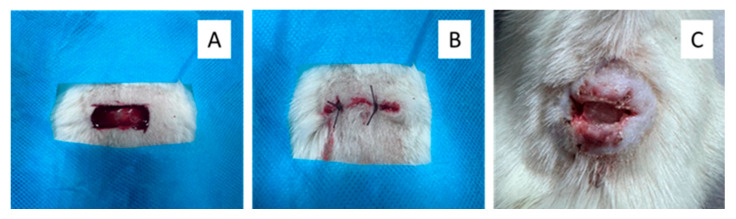
Macroscopic appearances of full-thickness skin defects modelling in rats: (**A**) wound formation, (**B**) introduction of a gauze swab with bacterial suspension into the deep layer of the skin, and (**C**) the appearance of a purulent wound after 3 days.

All laboratory animals were individually anesthetized via intravenous administration of medetomidine hydrochloride (“Prosedan”, Farmaton, Kyiv, Ukraine) at a dosage of 10 mg per kilogram of body weight. Before the experiment, the surgical field located in the interscapular region was carefully prepared by shaving, treating with 70% ethanol, and covering with a sterile cloth to ensure an aseptic condition [49]. A full-thickness wound measuring 1.0 cm × 1.5 cm (1.5 cm^2^) was then surgically created on the dorsum of each rat. To induce purulent inflammation, a sterile gauze swab soaked in a bacterial suspension containing *Staphylococcus aureus*, *Escherichia coli*, and *Pseudomonas aeruginosa* (5 × 10^9^ CFU/mL for each strain) was placed into the wound, and the site was sutured. After 72 h, once purulent inflammation had developed, the wound was reopened to remove the gauze swab and any residual pus (Figure 1).

To evaluate different wound treatment strategies, the animals were randomly divided into three groups (18 rats per group): (i) Ch/PLA, (ii) Ch/PLA-AgNPs, and (iii) PLA-chlorhexidine. A sterile experimental membrane with a size of 1.5 cm^2^, with and without AgNPs, was applied as described for the first and second groups, respectively, while the third group (control) received PLA membranes treated with a pharmaceutical 0.05% chlorhexidine solution (10 µL per sample).

Following the application of the wound treatment materials, a sterile occlusive dressing was applied to minimize the risk of cross-contamination from the skin surface. Daily dressing changes were performed under aseptic conditions in accordance with the principles of purulent surgery.

The animals were humanely euthanized at 3, 14, and 21 days post-treatment (6 animals per time point) using an overdose of medetomidine hydrochloride (“Prosedan”, Farmaton, Ukraine) at a dosage of 70 mg per kilogram of body weight. Subsequently, the total wound area was harvested for histological analysis to assess the effectiveness of the applied treatments.

### 2.5. Wound Size and Microbiology Profile Monitoring

The animals were observed daily, and digital images of the wounds were taken to monitor the healing progression. The wound area was measured by tracing the wound margins and calculating the pixel area using the open-access Image-J 1.52a software (Wayne Rasband, Bethesda, MD, USA).

Quantitative and qualitative assessments of wound microbial contamination were conducted using surface swab sampling of wound exudates. The first sample was collected on day 1 after purulent wound modeling, followed by additional samples collected on days 3, 5, and 7 of the experiment. Swabs were taken from both the central and peripheral regions of the wounds using sterile cotton swabs for further microbiological analysis. The samples were processed using the streak plate technique, with inoculation on selective agar media, including MacConkey agar, mannitol-salt agar, and cetrimide agar. The inoculated plates were incubated at 37 ± 1 °C for 24 h. Colony counts were subsequently performed and expressed in logarithmic form to quantify bacterial contamination.

### 2.6. Histology and Immunohistochemistry

The histological and immunohistochemical staining were performed as described before [50]. Briefly, tissue samples were fixed in 10% neutral buffered formaldehyde (Sigma-Aldrich, St. Louis, MO, USA). Following fixation, the samples were dehydrated with an ethanol gradient and saturated with paraffin in a tissue processor (AT1010-EKA, Mariupol, Ukraine). Paraffin blocks were embedded using the ES5-EKA embedding station (Mariupol, Ukraine), and serial sections, each 4 µm thick, were prepared with a Shandon Finesse 325 rotary microtome (Thermo Scientific, Waltham, MA, USA) and attached to SuperFrost adhesive microscopy slides (Thermo Scientific, Waltham, MA, USA). The sections were stained with Mayer’s hematoxylin and eosin and mounted using a permanent mounting medium (BioGnost, Zagreb, Croatia).

Immunohistochemical staining was performed using a Master polymer plus detection system (Peroxidase) (Master Diagnostica, Granada, Spain). Following protocol, the tissue sections were deparaffinized and dehydrated. Afterward, the samples were subjected to antigen retrieval by heat incubation at 98 °C in 10 mM citrate buffer (pH 6.0) for 30 min. Endogenous peroxidase activity was then blocked, and the samples were incubated with a blocking agent. After blocking, they were incubated with primary polyclonal antibodies: anti-CD68 (dilution 1:200, MyBioSource, San Diego, CA, USA), anti-CD163 (ready to use, Master Diagnostica, Granada, Spain), and anti-MPO (dilution 1:200, Thermo Scientific, Waltham, MA, USA). The tissue samples were later incubated with the HRP-polymer solution and visualized using an immunoperoxidase DAB kit (Master Diagnostica, Granada, Spain). The nuclei were counterstained with Mayer’s hematoxylin, and the samples were mounted with a mounting medium from Master Diagnostica (Granada, Spain).

## 3. Results

### 3.1. Patches Structure

**Figure 2:**
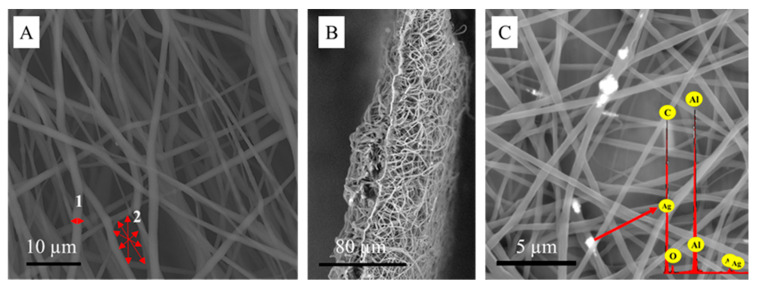
Scanning electron microscopy of (**A**) Ch/PLA membrane (1—average fiber diameter, 2—porous area fraction; red arrows indicate the direction of the measurements) with the cross-sectional view (**B**), (**C**) Ch/PLA-AgNPs membrane, and EDX analysis loaded with 100 µg of AgNPs(red arrow marks the point where the measurements were performed).

Both Ch/PLA and Ch/PLA-AgNPs membranes consisted of randomly oriented fibers forming interconnected pores, as shown in Figure 2. The fibers had an average diameter of 1.55 ± 0.78 μm. The membrane morphology displayed uniform, bead-free fibers with minimal thin fibers. The electrospun patches exhibited a porous area fraction of 9.38 ± 1.19% [48,49,51].

### 3.2. Wound Healing Rate

**Figure 3:**
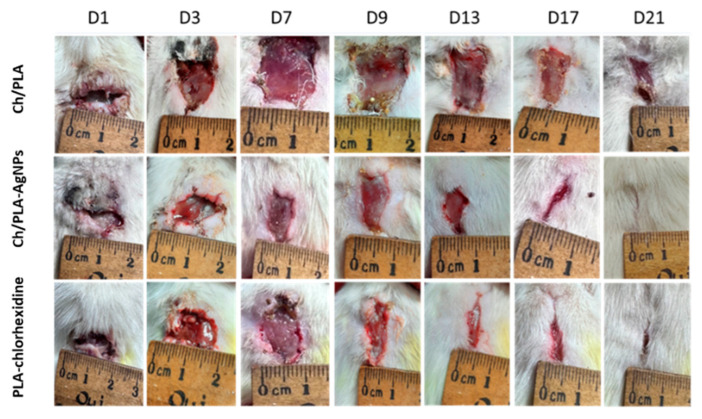
Representative images of full-thickness skin defects in rats in different groups.

Wound healing was quantitatively assessed by measuring the wound area, with closure and recovery evaluated based on the type of treatment applied (Figure 3). On day 1, the skin defects exhibited typical characteristics of purulent wounds. Initial signs of cleansing from pathological detritus were observed between days 5 and 6 in the groups treated with PLA-chlorhexidine and Ch/PLA membranes modified with AgNPs, whereas this process began later, on day 8, in animals treated with Ch/PLA membranes without silver nanoparticles.

Notably, epithelialization was achieved earlier, on day 15, in rats treated with Ch/PLA-AgNPs and PLA-chlorhexidine membranes, compared to day 17 in the group treated with Ch/PLA membranes alone. Morphometric analysis revealed that purulent inflammation caused an initial increase in wound area by day 4 of the experiment. No significant differences in wound area dynamics were observed among the treatment groups up to day 9. However, starting from day 10, a marked acceleration in wound closure was evident in the PLA-chlorhexidine and Ch/PLA-AgNPs groups.

**Figure 4:**
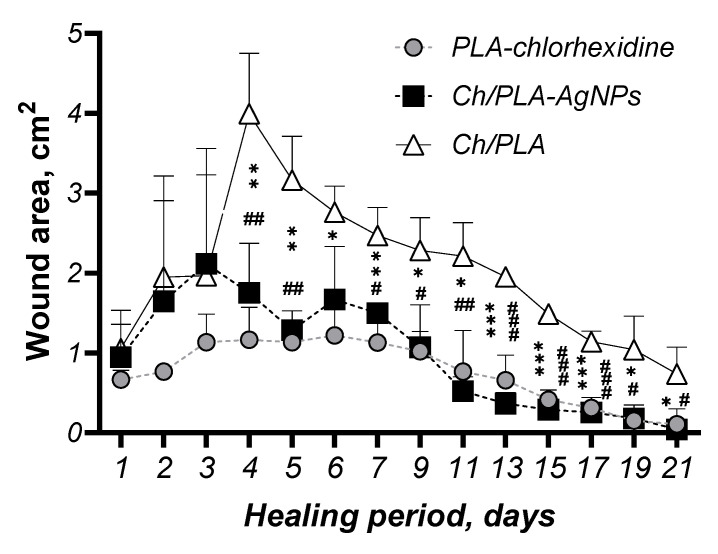
Wound size dynamics in a rat skin defect model after various treatments (*n* = 18). Statistical significance is indicated as follows: * *p* < 0.05, ** *p* < 0.01, *** *p* < 0.001 represent differences between Ch/PLA and PLA-chlorhexidine membranes; # *p* < 0.05, ## *p* < 0.01, ### *p* < 0.001 represent differences between Ch/PLA-AgNPs and Ch/PLA membranes.

The macroscopic examination of wounds provided insights into the healing process and the reduction in wound size at different time points during the experiment. Figure 4 illustrates the dynamics of wound closure across various days during the recovery phase. Consistent with the in vitro antimicrobial activity results, where the Ch/PLA-AgNPs samples exhibited the highest efficacy on days 5 and 7, the wound healing rate significantly reduced the affected surface area starting from day 5 in the groups treated with membranes containing silver nanoparticles. Additionally, Ch/PLA-AgNPs samples demonstrated a greater decrease in wound surface area compared to the group treated with non-modified Ch/PLA membranes. This enhanced performance can be attributed to the inherent healing properties of the chitosan membrane structure, further amplified by the antimicrobial and regenerative effects of the incorporated silver nanoparticles.

### 3.3. Wound Microbiology

**Figure 5:**
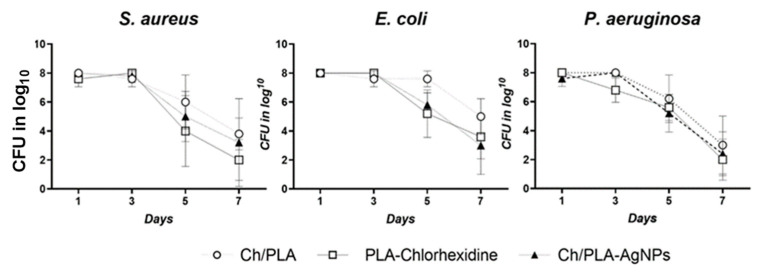
The qualitative microbiological composition of the wounds assessed at various time points throughout the treatment.

The microbiological profile of the experimental purulent wounds demonstrated that the applied treatments effectively eliminated bacterial populations within 4–6 days, depending on the bacterial species (Figure 5). By day 7 following the initiation of treatment, all methods resulted in a substantial reduction in bacterial colonization. Among the approaches, modifying CH/PLA membranes with AgNPs proved to be the most effective in eradicating bacteria.

Significant differences between the groups emerged on days 5 and 7. For *E. coli* and *S. aureus*, the bacterial counts were reduced to 5 log CFU/mL and 4 log CFU/mL, respectively, in the CH/PLA-AgNP-treated samples. Similarly, *P. aeruginosa* counts were reduced to 5 log CFU/mL and 4 log CFU/mL. Notably, the elimination of *E. coli* by these time points was comparable between the AgNP-loaded membranes and the control sample containing chlorhexidine, both achieving reductions to 5 log CFU/mL and 4 log CFU/mL, respectively.

Evaluating progressive wound changes during the healing process involves the application of techniques that analyze specific parameters. Effective wound assessment depends on understanding the healing pathophysiology, identifying factors that delay the process, and establishing optimal conditions at the wound bed to enhance healing and therapeutic effectiveness. It is essential to conduct and document a thorough analysis of the healing progression of both the wound and the surrounding tissue during follow-up [52].

### 3.4. Histological and Immunohistochemical Assessment

Histological evaluation and grading of purulent wound healing provide a direct and reliable method for evaluating the effectiveness of dressing materials, particularly those made from different compositions. In this study, we investigated three types of dressing materials: polylactic acid polymer combined with chitosan nanofibers (Ch/PLA-nanofiber), a complex of silver nanoparticles integrated with polylactic acid and chitosan nanofibers (Ch/PLA-AgNPs), and a combination of polylactic acid with chlorhexidine (PLA-Chlorhexidine). The experimental observations were conducted on days 3, 14, and 21 in all three groups of rats.

**Figure 6:**
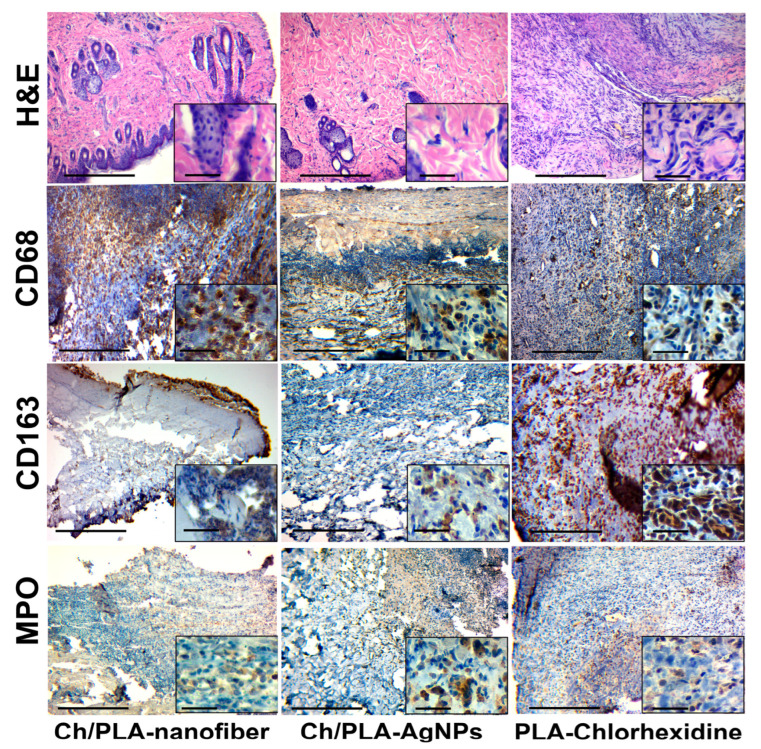
Histological and immunohistochemical evaluation of the skin samples from experimental animals on the third day of the treatment. The scale bar corresponds to 200 µm for the main image and 25 µm for the inset.

Treatment with the Ch/PLA-nanofiber dressing resulted in wounds filled with necrotic detritus and remnants of the dressing material on the third day (Figure 6). The wound was confined and surrounded by extensive neutrophilic inflammatory infiltration. Histologically, the bottom of the wound was primarily represented by the hypodermis. Adjacent tissues exhibited interstitial edema across all skin layers, while the epidermis near the wound demonstrated dystrophic changes in the covering epithelium.

In the group treated with Ch/PLA-AgNPs dressings, the wound area contained moderate necrotic detritus during the first observation period. The wound contents also displayed moderately expressed fibrinous-purulent exudation and neutrophilic inflammatory infiltration. Additionally, we observed the formation of immature granulation tissue with moderate inflammatory infiltration, along with remnants of dressing material. These remnants, stained with hematoxylin, were characteristic of chitosan.

The use of PLA-Chlorhexidine dressing material resulted in wounds filled with necrotic tissues, which were surrounded by an «inflammatory shaft» composed of neutrophils and lymphocytes on the third day. Remnants of the dressing biomaterial and a «scab» were visible within the wound detritus. The wound wall and bottom contained immature granulation tissue with diffuse inflammatory infiltration. The hypodermis, representing the reticulated layer of the dermis, formed the wound’s base and showed a spread of inflammatory infiltrate between the connective tissue fibers. Edema and signs of exudation were also evident in the surrounding skin layers.

**Figure 7:**
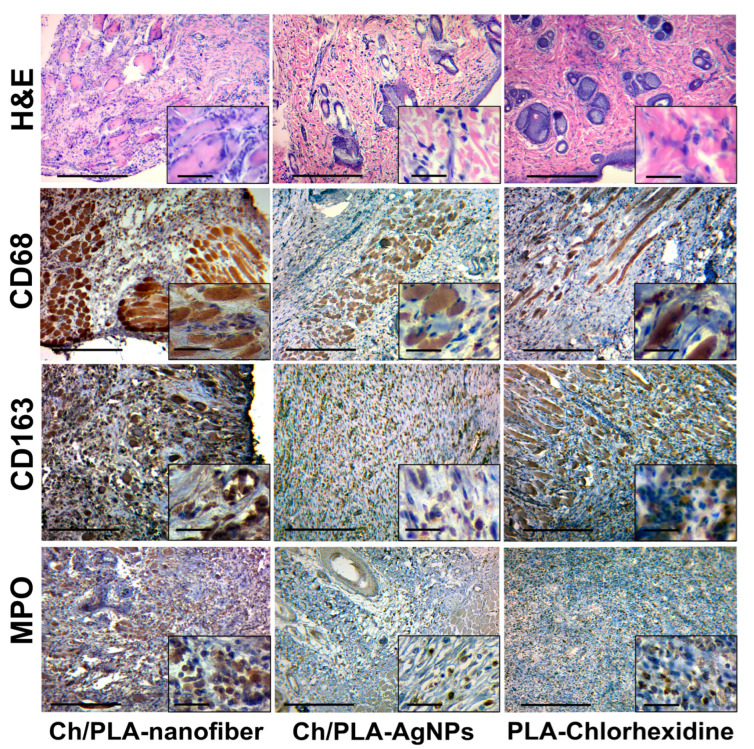
Histological and immunohistochemical evaluation of the skin samples from experimental animals on the 14th day of the treatment. The scale bar corresponds to 200 µm for the main image and 25 µm for the inset.

Granulation tissue of varying degrees of maturity, accompanied by signs of inflammatory infiltration and edema, was observed after 14 days of treatment with the Ch/PLA-nanofiber dressing material (Figure 7). The epidermis covering the wound exhibited dystrophic changes, while the inflammatory process was characterized by a diffuse, non-intense inflammatory infiltrate. This infiltrate spread between the fibers of the dermis and was primarily composed of lymphocytes and histiocytes, with an admixture of fibroblasts. Edema and dyscirculatory disorders were evident across all skin layers.

In the group treated with Ch/PLA-AgNPs dressing material, mature granulation tissue predominated in the wound after 14 days. Minimal inflammatory infiltration of mixed lymphohistiocytic composition was observed, accompanied by minor edema and fibrillization (sclerosis), indicating an increase in connective tissue fibers in the dermis. Additionally, large granular cells of irregular shape, likely representing degranulating neutrophils or activated fibroblasts, were noted in the adjacent tissues.

A significant amount of granulation tissue with varying degrees of maturity was detected during the second control period for wounds treated with PLA-Chlorhexidine dressing material. Diffuse inflammatory lymphohistiocytic infiltration was present, with neutrophils spreading into the skin tissue adjacent to the wound defect. Numerous neutrophils with signs of degranulation were identified, forming the “outer border” of the wound process and reflecting ongoing immune activity.

**Figure 8:**
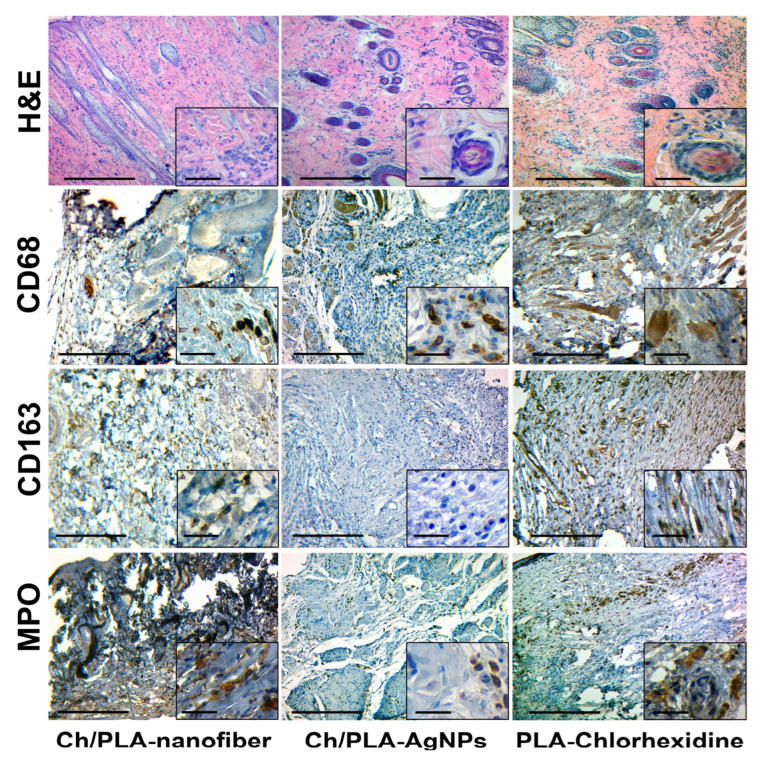
Histological and immunohistochemical evaluation of the skin samples from experimental animals on the 21st day of the treatment. The scale bar corresponds to 200 µm for the main image and 25 µm for the inset.

Skin samples treated with the Ch/PLA-nanofiber dressing exhibited edema across all layers, coarsening of collagen and elastic fibers in the papillary layer, and optically empty spaces around hair follicles and sebaceous glands at the final control period (Figure 8). The tissue contained a significant amount of connective tissue and remnants of mature granulation tissue, accompanied by weak but diffuse inflammatory infiltration. The inflammatory infiltrate was predominantly composed of lymphocytes and histiocytes.

After 21 days of treatment with the Ch/PLA-AgNPs dressing, the skin tissues demonstrated fibrous changes with minimal inflammatory infiltration. The infiltrate consisted of individual cells and clusters of lymphocytes. The tissues contained fully blood-filled vessels and moderate edema. Most samples displayed unidirectional positive changes, characterized by remnants of mature granulation tissue and moderate inflammatory infiltration, indicating progressive healing.

For wounds treated with the PLA-Chlorhexidine dressing, the defect was replaced by fibrous tissue with mostly mild diffuse inflammatory mixed-cell infiltration by the 21st day. The infiltrate was predominantly lymphocytic, with isolated macrophages, plasma cells, and neutrophils. Hemosiderophages were identified, and mild edema was present across all skin layers within the connective tissue fibers, further reflecting an advanced stage of wound recovery.

The skin tissues of rats with a simulated purulent wound process are characterized by inflammatory cell infiltration. This typical pathological process can be assessed qualitatively and quantitatively by immunohistochemical evaluation of pro- (M1) and anti-inflammatory (M2) macrophage markers. The activity and acute phase of inflammation are characterized by the number of neutrophils in the inflammatory infiltrate.

The immunohistochemical marker CD68 has a cytoplasmic staining in the cell, which is characterized by macrophages of the pro-inflammatory phenotype M1, which perform a protective and scavenging function [53]. Also, this marker stains (overlapping) a part of fibroblasts and endotheliocytes [54]. A large number of M1-type macrophages are found among detritus, necrotized tissue, immature granulation tissue, and inflammatory infiltrates in the wound tissue.

**Figure 9:**
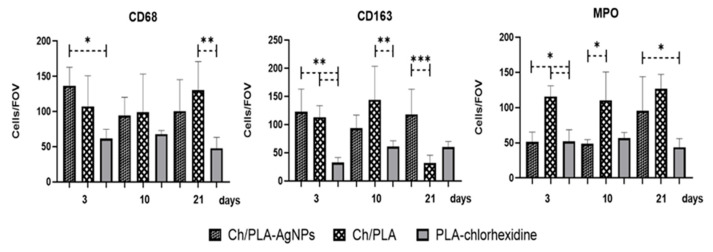
Immunohistochemical evaluation of the cells in the skin samples from experimental animals. Skin tissue samples were assessed immunohistochemically with further quantitative evaluation of CD68+, CD163+, and MPO+ immune cells at the different time points of the treatment. * *p* < 0.05, ** *p* < 0.01, *** *p* < 0.001.

The CD163 marker also has cytoplasmic staining and is used to identify macrophages of the anti-inflammatory M2 phenotype. This phenotype has an immunosuppressive and proliferative effect and is a permanent element of the tumor microenvironment [53]. It is expected that the number of M2 phenotype macrophages will increase with the strengthening of reparative processes and a decrease in the intensity (suppression) of inflammation [55]. The immunohistochemical study of CD68- and CD163-positive cell expression has limitations and caveats. The main problem is the positive overlapping by these markers of some cell populations, such as fibroblasts, endotheliocytes, and pericytes [56]. Marker MPO, or myeloperoxidase, is the main enzyme of neutrophils and is used for their immunohistochemical identification [57]. A large number of neutrophils is a direct sign of acute purulent inflammation. Therefore, the presence of even a small number of neutrophils in the inflammatory infiltrate is a sign of the activity of the inflammatory process. The number of CD68-positive cells in the group treated with Ch/PLA-AgNPs dressing material was high at the beginning of the experiment and subsequently decreased to moderate values. The dynamics of CD163-positive cells in the tissues of the purulent wound showed the following trend: we observed a relatively large number of M2 macrophages, followed by their moderate decrease until the end of the experiment. Interestingly, the number of neutrophils (MPO-positive cells) in the Ch/PLA-AgNPs group increased in the wound towards the end of the experimental study (Figure 9).

The number of pro-inflammatory M1 macrophages in the Ch/PLA dressing material group increased from the beginning to the end of the experiment, while the number of M2 macrophages increased by week 2, with a further decrease at the end of the experiment. Inflammatory activity was high throughout the experiment and continued to increase according to the number of MPO-positive cells.

In the skin samples from animals, treated with the PLA-Chlorhexidine dressing material, the number of CD68-positive M1-type macrophages was at a medium to low level at the end of the experiment. The number of M2-type macrophages increased slightly at the end of the treatment. The level of neutrophils in the PLA-Chlorhexidine-treated group was low.

### 3.5. Systemic Reaction for Biomaterials Application

We performed a histological analysis of the experimental animals’ internal organs to evaluate the effect of using a dressing material based on polylactic acid polymer and the addition of components such as chitosan nanofibers, silver nanoparticles, and chlorhexidine.

**Figure 10:**
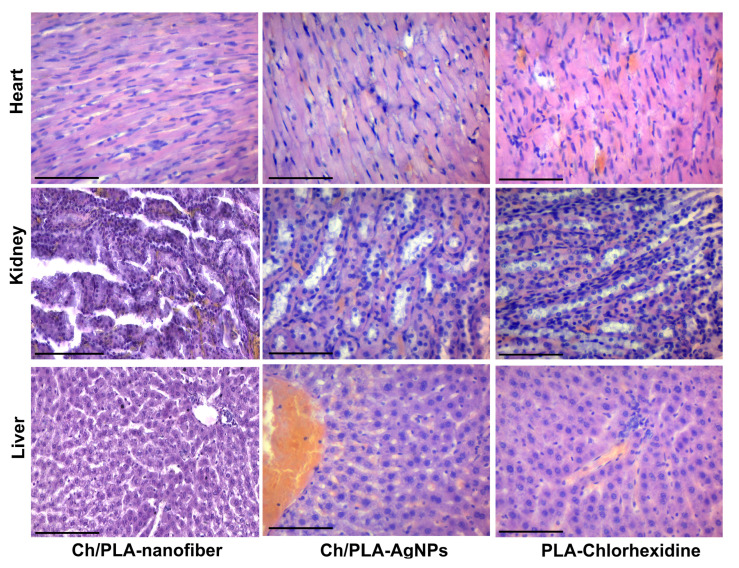
Histological evaluation of internal organs after application of different dressing materials for infected wound healing on the third day after treatment initiation. The magnification corresponds to ×400 (the scale bar is equal to 100 μm).

On the third day of the experiment, a histological evaluation of rats’ tissue samples, treated with a Ch/PLA dressing material in the heart, did not reveal significant structural disorders: the myocardial tissue contained signs of edema, interfibrous spaces, and capillary plethora (Figure 10). The kidney tissue also had a preserved histological structure with minimal dyscirculatory changes as uneven blood supply and small hemorrhages. Signs of venous congestion, granularity of hepatocytes, and swelling of the interlobular spaces were found in the liver. The Ch/PLA-AgNPs dressing material used to treat purulent wounds showed no significant morphological changes on the third day of the experiment. Thus, the tissue of the myocardium had a preserved histostructure with minor edema and fullness of the capillaries. Morpho-functional changes in kidney and liver tissue were limited to minor microcirculation disorders. In general, the parenchymal organs of experimental animals did not have dystrophic changes. The PLA-Chlorhexidine dressing material left minor changes in the internal parenchymal organs after 3 days of purulent skin infection treatment. Thus, we found small hemorrhages and edema in the interfibrous spaces in the myocardium. The tissue of the kidneys and liver contained capillary plethora and edema of the epithelial tissues.

**Figure 11:**
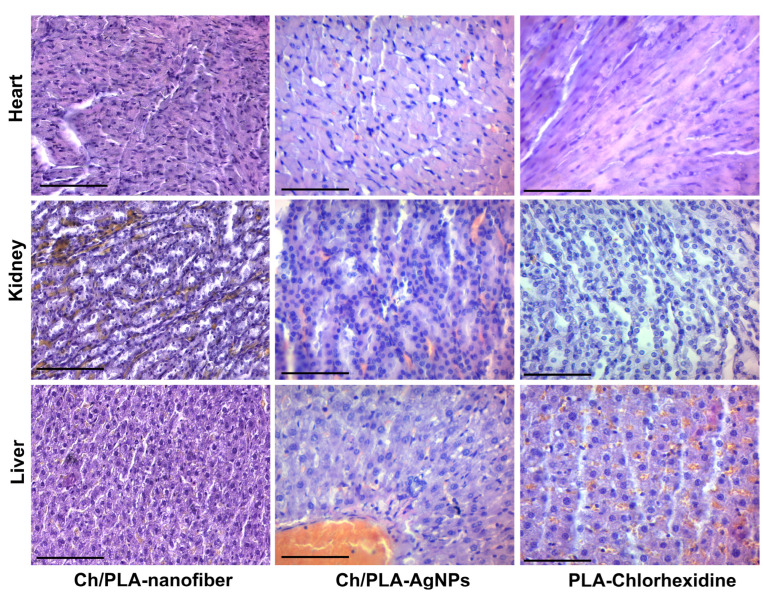
Histological evaluation of internal organs after application of different dressing materials for infected wound healing on the 14th day after treatment initiation. The magnification corresponds to ×400 (the scale bar is equal to 100 μm).

We found moderate changes in the heart after 14 days of treatment with Ch/PLA dressing material (Figure 11). Myocardial tissue contained cardiomyocytes with signs of fine droplet fatty dystrophy, stroma swelling, and engorgement of small vessels. The kidney tissue had distinct microcirculation disorders such as capillary plethora, small hemorrhages, and edema. The liver tissue contained signs of protein dystrophy in individual groups of hepatocytes. Erythrocytes and lymphocytes were found in the swollen interstitial spaces.

On the 14th day, we observed the presence of moderate dyscirculatory changes in the form of pleurisy of small vessels, sludge in individual capillaries, and swelling of interfibrous spaces in the myocardium of experimental animals, treated with Ch/PLA-AgNPs dressing material. The tissue of the kidneys and liver had a preserved structure. Thus, we found only the granularity of nephrothelium and hepatocytes and minor microcirculatory disorders of the tissues. The use of PLA-Chlorhexidine dressing material for 2 weeks showed the presence of separate histological changes, mainly in the liver tissue. This tissue contained capillary engorgement, swelling of the interlobular spaces, and signs of moderate dystrophic changes in hepatocytes. Myocardium and kidney tissue had only minor dyscirculatory changes.

**Figure 12:**
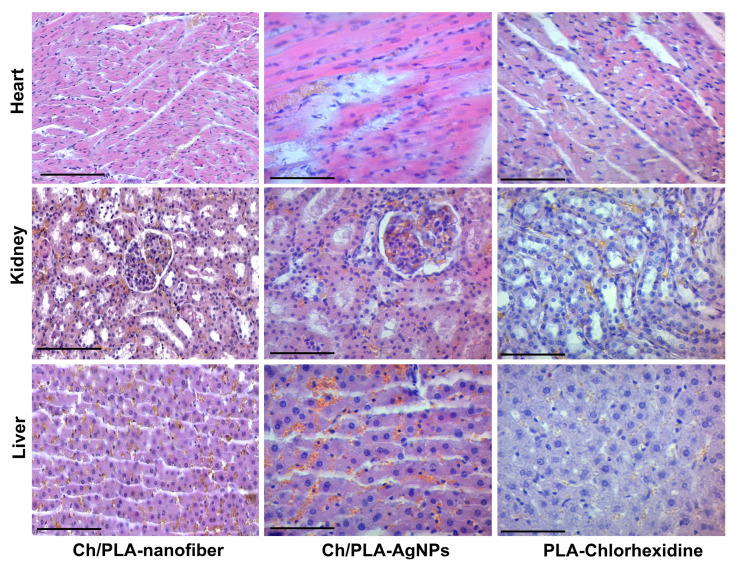
Histological evaluation of internal organs after application of different dressing materials for infected wound healing on the 21st day after treatment initiation. The magnification corresponds to ×400 (the scale bar is equal to 100 μm).

At the end of the treatment, i.e., after 21 days, we noted individual histological transformations of the studied tissues in all the studied groups (Figure 12). Thus, the heart tissue of animals, treated with Ch/PLA dressing material, contained distinct signs of a tissue microcirculation disorder—hemorrhaging of medium and small vessels, stroma swelling, and areas of hemorrhage. The kidney tissue had widespread edema of the stroma, dilatation and fullness of capillaries according to the type of «blood lakes», and signs of moderate dystrophy of the glomerular epithelium. The liver tissue had venous congestion, hemorrhages in the interstitial spaces, and signs of fatty dystrophy of certain groups of hepatocytes.

The heart tissue of the experimental animals, treated with Ch/PLA-AgNPs dressing material, had uneven blood supply and coloration, edema, and capillary congestion in the stroma on the 21st day of the experiment. The kidney tissue contained dyscirculatory signs in the form of vessel plethora, hemorrhages in the intertubular stroma, and marked edema. We noted minor protein dystrophy phenomena of the glomeruli and tubules’ epithelium. There was a venous plethora, erythrocytes in the interlobular spaces, and small droplet fatty dystrophy of hepatocytes in the tissue of the experimental animals.

In the heart tissue of the animals that were treated with PLA-Chlorhexidine dressing material, after 21 days of the experiment, we found uneven coloring of the myocardium, noticeable edema with the formation of optical cavities in the tissue, and capillary plethora. During the same period, we found the granularity of the tubular epithelium, the presence of secretions and single desquamated cells in the lumen of the tubules, and general swelling in the kidney tissue. Liver tissue contained moderate protein and small droplet fatty dystrophy of hepatocytes, the interlobular spaces are swollen, containing erythrocytes and single lymphocytes.

## 4. Discussion

The findings of this study highlight the significant potential of Ch/PLA-AgNPs electrospun membranes as an advanced solution for the treatment of purulent wounds. In our previous research, we demonstrated the technology of Ch/PLA scaffold development and AgNPs immobilization and showcased their effectiveness and safety in experiments in vitro [47,48]. The current in vivo study demonstrated that incorporating silver nanoparticles into the Ch/PLA matrix enhanced the antimicrobial efficacy and promoted faster wound closure and tissue regeneration compared to conventional treatments, such as PLA membranes treated with chlorhexidine or Ch/PLA membranes without modifications.

One notable advancement of our Ch/PLA-AgNPs technology lies in its superior wound-healing performance. By day 15, wounds treated with the AgNP-modified membranes exhibited complete epithelialization, surpassing the healing rates observed in non-modified Ch/PLA membranes and equaling or exceeding those treated with PLA-chlorhexidine membranes. This result underscores the synergistic effect of combining chitosan’s biocompatibility and regenerative properties with the potent antimicrobial activity of silver nanoparticles.

In comparison to existing technologies, such as silver sulfadiazine or other nanoparticle-based treatments, our Ch/PLA-AgNPs membranes offer distinct advantages in terms of both functionality and safety [58]. Unlike silver sulfadiazine, which has been associated with cytotoxicity and delayed wound healing in some cases [59,60], the Ch/PLA-AgNPs membranes exhibited high biocompatibility with no signs of systemic toxicity in adjacent tissues or internal organs during this study. Furthermore, the electrospun architecture of the membranes provides a high surface area-to-volume ratio [61], allowing for optimal oxygen exchange and exudate absorption, features that are critical in managing infected wounds [62].

The antimicrobial performance of Ch/PLA-AgNPs membranes also proved superior, with significant bacterial load reductions observed by day 5 post-treatment. Comparative studies have shown that traditional chitosan-based materials struggle to eliminate biofilm-associated bacteria without the addition of potent antimicrobial agents [63,64,65]. The inclusion of silver nanoparticles in our composite effectively addresses this limitation, achieving rapid and sustained bacterial clearance, even against pathogens embedded in biofilms [66]. Additionally, electrospinning as the production method enables precise control over fiber morphology, porosity, and mechanical properties. This customization facilitates the creation of membranes tailored to the complex microenvironments of purulent wounds, outperforming conventional solvent-cast or freeze-dried dressings that lack comparable nanostructural features.

Histological analysis of the possible toxic effect on the experimental animals’ internal organs under the treatment of purulent wounds with dressing material based on polylactic acid polymer and adding chitosan nanofibers, silver nanoparticles, and chlorhexidine showed its insignificant effect. The main changes related to microcirculation disorders include edema, congestion of small and medium vessels, and minimal and moderate dystrophic changes. Although most of the histological transformations of tissues of parenchymal organs can be explained by the conditions of taking tissues for research and the presence of stress in experimental animals, it is still necessary to note the better morpho-functional state and preservation of the internal organs’ parenchyma in the group treated with Ch/PLA-AgNPs dressing material.

Changes in the internal organs of the group treated with Ch/PLA dressing material can be explained by general intoxication against the background of purulent infection, since there was no additional antibacterial effect. The group of animals treated with PLA-Chlorhexidine had more pronounced changes in the liver, with a more preserved structure of the heart and kidneys. This result can be explained by the effect of chlorhexidine, which is to some extent metabolized by the liver and excreted with bile [67]. According to the immunohistochemical data, the most optimal results were observed in the animal group, treated with PLA-Chlorhexidine dressing material. In this group, the level of inflammation was low, and the ratio of macrophage types M1/M2 was consistent with the logical course of the wound process, with the predominance of pro-inflammatory cells at the beginning, followed by reparative changes at the end of the experiment. These results can be explained by the presence of the antibacterial effect of chlorhexidine, which contributed to the timely cleaning and healing of the wound. The Ch/PLA dressing material showed a high level of inflammatory activity and a reduced level of reparative processes. These results can be explained by the absence of a distinct antibacterial component in the composition of the dressing material. The Ch/PLA-AgNPs dressing material showed encouraging results in controlling the inflammatory process. At the same time, it showed a certain imbalance in the reparative component of wound healing. A possible reason may be the presence of chitosan fibers, which affect macrophage activity, as they require degradation, and this disrupts the M1/M2 balance of macrophages during the wound healing process. Further research is needed to clarify the mechanism of the effect of chitosan in the composition of the combined dressing material.

Overall, this study demonstrates that Ch/PLA-AgNPs membranes significantly advance wound care technology. By combining chitosan, PLA, and silver nanoparticles into a single electrospun scaffold, we address the multifaceted challenges of infected wounds, including microbial colonization, biofilm resistance, and impaired tissue regeneration. Future studies should focus on scaling production and conducting clinical trials to further validate the efficacy and safety of these membranes in diverse patient populations.

## 5. Conclusions

The results of this study provide strong evidence for the effectiveness of Ch/PLA-AgNPs electrospun membranes in promoting the healing of purulent wounds. By leveraging the biocompatibility and regenerative properties of chitosan, the structural stability of PLA, and the potent antimicrobial activity of silver nanoparticles, this novel biomaterial demonstrated superior performance in both bacterial eradication and wound closure compared to existing treatment options.

The in vivo experiment confirmed that the Ch/PLA-AgNPs membranes significantly accelerated the healing process, achieving complete epithelialization by day 15, surpassing the performance of both non-modified Ch/PLA membranes and PLA-chlorhexidine controls. Moreover, the membranes effectively reduced bacterial colonization within 4–6 days, even in wounds with high microbial loads and biofilm-associated pathogens, such as *S. aureus*, *P. aeruginosa*, and *E. coli*. This antimicrobial efficacy was achieved without any observable systemic toxicity, highlighting the safety and therapeutic potential of the material.

Importantly, the advanced electrospun architecture of the membranes provided a tailored environment for tissue regeneration, optimizing oxygen permeability, exudate absorption, and mechanical support. These features, combined with the demonstrated effectiveness in vivo, position the Ch/PLA-AgNPs membranes as a significant step forward in the development of advanced wound care technologies.

This study underscores the importance of rigorous evaluations in vivo in bridging the gap between laboratory findings and clinical applications. By addressing critical challenges in wound healing, including biofilm resistance and tissue regeneration, the Ch/PLA-AgNPs membranes provide a promising platform for future clinical translation.

Nevertheless, further investigations are needed to optimize the formulation for long-term biocompatibility and clinical translation. Particular attention should be given to (i) the long-term biodistribution and clearance of AgNPs; (ii) potential cumulative effects of chronic exposure to chitosan and silver components on internal organs; and (iii) understanding how the composite modulates macrophage polarization and inflammation resolution over extended periods. These studies are essential to minimize translational risks and maximize therapeutic benefits.

## Data Availability

The original contributions presented in this study are included in the article. Further inquiries can be directed to the corresponding author.
